# Quantitative Assessment of Collagen Architecture to Determine Role of Tumor Stroma During Vestibular Schwannoma Progression

**DOI:** 10.1002/ohn.1018

**Published:** 2024-11-07

**Authors:** Melanie Fisher, Bailey H. Duhon, Han T. N. Nguyen, Jeffrey R. Tonniges, Kyle C. Wu, Yin Ren

**Affiliations:** 1 Department of Otolaryngology–Head and Neck Surgery, Division of Otology, Neurotology and Cranial Base Surgery, The Ohio State University Wexner Medical Center, Columbus, Ohio, USA; 2 Campus Microscopy and Imaging Facility, Comprehensive Cancer Center, The Ohio State University, Columbus, Ohio, USA; 3 Department of Neurosurgery, The Ohio State University Wexner Medical Center, Columbus, Ohio, USA

**Keywords:** collagen, picro-sirius red, tumor microenvironment, vestibular schwannoma

## Abstract

**Objective.:**

The primary objective was to characterize the abundance and architecture of collagen in the extracellular matrix in vestibular schwannoma (VS). The secondary objective was to investigate the association between collagen architecture and tumor size.

**Study Design.:**

Retrospective cohort study.

**Setting.:**

Academic referral center.

**Methods.:**

Tumor samples were obtained from patients with sporadic VS undergoing microsurgical resection. Histological analyses were performed including picrosirius red (PSR) staining under polarized light. Collagen architecture was quantified using an automated fiber detection software. Second Harmonic Generation (SHG) microscopy and immunofluorescence (IF) were utilized to characterize collagen architecture.

**Results.:**

Eleven tumor specimens were included (mean tumor diameter = 2.80 cm, range 1.5–4.0 cm), and were divided into large (mean diameter = 3.5 ± 0.4 cm) and small (mean tumor diameter = 2.0 ± 0.4 cm) cohorts based on size. The large VS cohort showed significantly higher collagen density (27.65% vs 12.73%, *P* = .0043), with more thick fibers (mature Type I, 24.54% vs 12.97%, *P* = .0022) and thin fibers (immature Type I or mature Type III, 23.55% vs 12.27%, *P* = .026). Tumor volume correlated with greater degree of collagen fiber disorganization (*P* = .0413, *r*^2^ = 0.298). Specifically, collagen type I intensity was significantly higher in large VS compared to small tumors (*P* < .001) and peripheral nerve (*P* = .028).

**Conclusion.:**

Larger VS exhibit increased collagen abundance in the tumor stroma, and a more disorganized collagen architecture compared to smaller VS and normal peripheral nerve tissue. This finding indicates that collagen organization may play a significant role in extracellular matrix remodeling and the progression of VS.

Vestibular schwannomas (VS) are tumors arising from Schwann cells lining the vestibulocochlear nerve (CN VIII).^1^ The tumor is intimately associated with nerves responsible for critical functions including hearing and balance. Tumor growth can lead to morbidities including hearing loss, tinnitus, imbalance, and vertigo. When tumors enlarge significantly, hydrocephalus and brainstem compression can result.^1^ The difficulty in managing VS is partly due to its unpredictable growth rate. Surgical resection is more challenging for certain VS subtypes that exhibit more aggressive behavior, such as tumors that have large cystic components, display significant adherence to surrounding cranial nerves and brainstem, and have elevated biomechanical stiffness.^2–4^ Understanding how changes in tumor stroma influence schwannoma progression is crucial for developing more accurate diagnostics and effective therapies, ultimately improving patient outcomes.

Changes in the tumor microenvironment (TME) are a hallmark of VS progression, significantly influencing tumor biomechanical stiffness and interactions with surrounding tissue.^5,6^ These changes are often driven by the interplay between tumor cells and extracellular components, with collagen playing a vital role due to its structural and signaling function in the TME.^5–7^ The increase in deposition and degradation of collagen fibers is regulated by diverse biomechanical^8^ and cytokine signaling pathways,^9,10^ leading to both an increased amount of collagen and more misaligned collagen fibers as the tumor grows. This dense and disorganized collagen not only stiffens the TME, facilitating the expansion and invasion of tumor cells,^11^ but also disrupts immune cell infiltration and response^6,12^ and contributes to therapy resistance.^13^ Additionally, specific collagen subtypes, such as Type I in breast cancer,^14^ Type III in colorectal cancer,^15^ and Type IV in pancreatic cancer^16^ have been shown to contribute to tumor progression. However, the role of collagen deposition and architecture in tumor progression has yet to be studied in VS.

Recently, multiomic analyses including single-cell RNA sequencing and proteomics have identified an upregulation of genes involved in extracellular matrix (ECM) remodeling. Specifically, these analyses highlighted collagen as a potential biomarker of VS by showing that elevated expression of specific collagen types are indicators of disease progression and treatment response.^17–19^ Moreover, enzymes responsible for collagen degradation, such as matrix metalloproteases (MMPs), have been studied as biomarkers for aggressive VS subtypes that are adherent, stiff, or cystic. Activated fibroblasts have also been shown to contribute to increased tumor stiffness by producing ECM components.^3,20^ Additionally, anti-fibrotic pharmacotherapies such as losartan, which aim to alleviate tumor fibrosis, was effective in rescuing hearing loss in a schwannoma mouse model. However, losartan did not significantly reduce tumor growth or hearing loss in other studies.^21,22^ These inconsistent results highlight the need for further investigation into the relationship between collagen and VS, as understanding these dynamics could be crucial for developing more effective treatments. Despite this, the specific role of collagen topology/architecture and its contribution to VS progression and growth have not been fully elucidated.

This study aims to advance our understanding of the relationship between collagen and VS by utilizing new staining methodology and objective measures to assess collagen deposition and accurately pinpoint specific collage subtypes. Additionally, we aim to assess collagen architecture by employing an automated image analysis platform. We hypothesize that as the tumor volume increases, remodeling of the TME leads to an increase in collagen deposition and an increase in misaligned collagen fibers, which may result in increased stiffness and symptomatic progression. Elucidation of this component of the TME may provide future insights into mitigating growth in aggressive VS and provide insight into potential therapeutic avenues.

## Methods

### Patient Samples and Tumor Processing

Formalin-fixed, paraffin-embedded (FFPE) VS tissue was sourced retrospectively from the archived tissue bank at the Ohio State University for samples collected between 2010 and 2023. All samples used in the study had appropriate institutional review board (IRB) approval from the Ohio State University Wexner Medical Center (IRB#1994H0241) and informed consent was obtained from all study participants. Normal Human Peripheral Nerve (HuFPT146) was obtained from Tissue Array. Clinical data collected included age, sex, tumor diameter, and follow-up information. To establish size cohorts, the average tumor diameter was calculated, and tumors were grouped into two categories, below the average (n = 5) and above the average (n = 6). Tumor volume was calculated using the ellipsoid formula 4/3 × π × 1/8(W × L × H), where W, L, and H represent the width, length, and height of the tumor in all 3 axes.

### Histological Staining

FFPE slides were deparaffinized in xylene, rehydrated in decreasing ethanol washes, and stained with Hematoxylin and Eosin (H&E), Masson’s Trichrome, or Picro Sirius Red (PSR). For PSR, sections were stained using Picro Sirius Red Stain Kit (Abcam ab150681) according to the manufacturer’s protocol. Briefly, sections were incubated in Picro Sirius Red Solution for 60 minutes, rinsed in acetic acid solution for 2 minutes, and dehydrated in absolute alcohol. Images were taken using a Zeiss Axioskop Microscope with a 4X/objective, a polarizer and analyzer for polarized microscopy, and a software controlled CCD camera with RGB filter (QImaging).

### Immunofluorescence Staining and Analysis

After deparaffinization and rehydration, paraffin sections underwent antigen retrieval and were blocked with 5% Donkey Serum for 1 hour at room temperature. Tissues were incubated in primary antibodies COL-1 (1:500, MA1–26771; Invitrogen) and S100 (GA504; Agilent Technologies) overnight at 4°C. Following primary antibody incubation, slides were stained with the appropriate secondary antibody and counterstained with 4′,6-diamidino-2-phenylindole (DAPI). Images were captured using an Olympus FV3000 laser scanning confocal microscope with a 20X/0.5 NA UPlanFL N objective and FluoView software (v2.6, Olympus Scientific). To determine collagen intensity per nuclei, Z-stacks were transformed into max intensity projections, split into blue and red channels, and converted to grayscale. After thresholding, the analyze particles function (0–100 μm^2^) was used on the blue channel to count the number of cells. The red channel was thresholded to remove the background in order to determine the collagen fluorescence intensity. This analysis was performed on 5 ROIs per sample.

### Collagen Quantification and Analysis

Collagen architecture was quantified by using an automated collagen fiber detection software, CT-Fire (Laboratory for Optical and Computation Instrumentation, University of Wisconsin). Polarized PSR images were converted into grayscale images using ImageJ and input into CT-Fire software using default settings. Results represent 2000 to 6000 fibers from combined results of ≥3 ROIs for each sample. Disorganization was defined as the percent of fibers ±20° from the median angle. The percentage of fibers was determined by dividing the number of fibers ±20° within the median by the total number of fibers. To determine collagen density and type, ≥3 polarized images for each sample were processed using ImageJ. First, images were converted to grayscale and split into red and green channels to differentiate between collagen subtypes. Both channels were thresholded to measure the percentage area of collagen and subtypes.

### Second Harmonic Generation Microscopy

Images were taken with an Olympus FV1000 multiphoton laser scanning microscope equipped with a MaiTai DeepSee tunable infrared laser (Spectra Physics) set to 910 nm and a ×10/0.40 NA UPlanXApo dry objective (Olympus Scientific). FFPE tissues were imaged directly without a glass coverslip. The backward scattered second harmonic generation signal was collected using the following detection wavelengths, 420 to 460 nm. Images were acquired with the FluoViewFV10-ASWv 4.2 (Evident, formally Olympus Scientific) software with 1.242 μm pixel size, and image tiling was performed by the multi area time lapse (MATL) function.

### Microarray Data Analysis

Microarray datasets (GSE39645 and GSE108524) for VS were analyzed for mRNA expression of collagen subtypes. Dataset GSE39645 consisted of 28 sporadic VS and 8 vestibular nerves, and Dataset GSE108524 consisted of 10 sporadic VS and 4 vestibular nerve controls.

### Statistical Analysis

Data were analyzed using GraphPad Prism 9.5.0. For binary comparisons, a 1-tailed Mann-Whitney *U* test was employed. Pearson’s correlation test was used to assess correlations. To compare multiple groups, a 1-way analysis of variance (ANOVA) was performed, followed by Tukey’s post hoc test for pairwise comparisons. *P* < .05 were considered statistically significant.

## Results

### Gene Expression of Several Subtypes of Collagen are Enriched in VS

**Figure 1.**
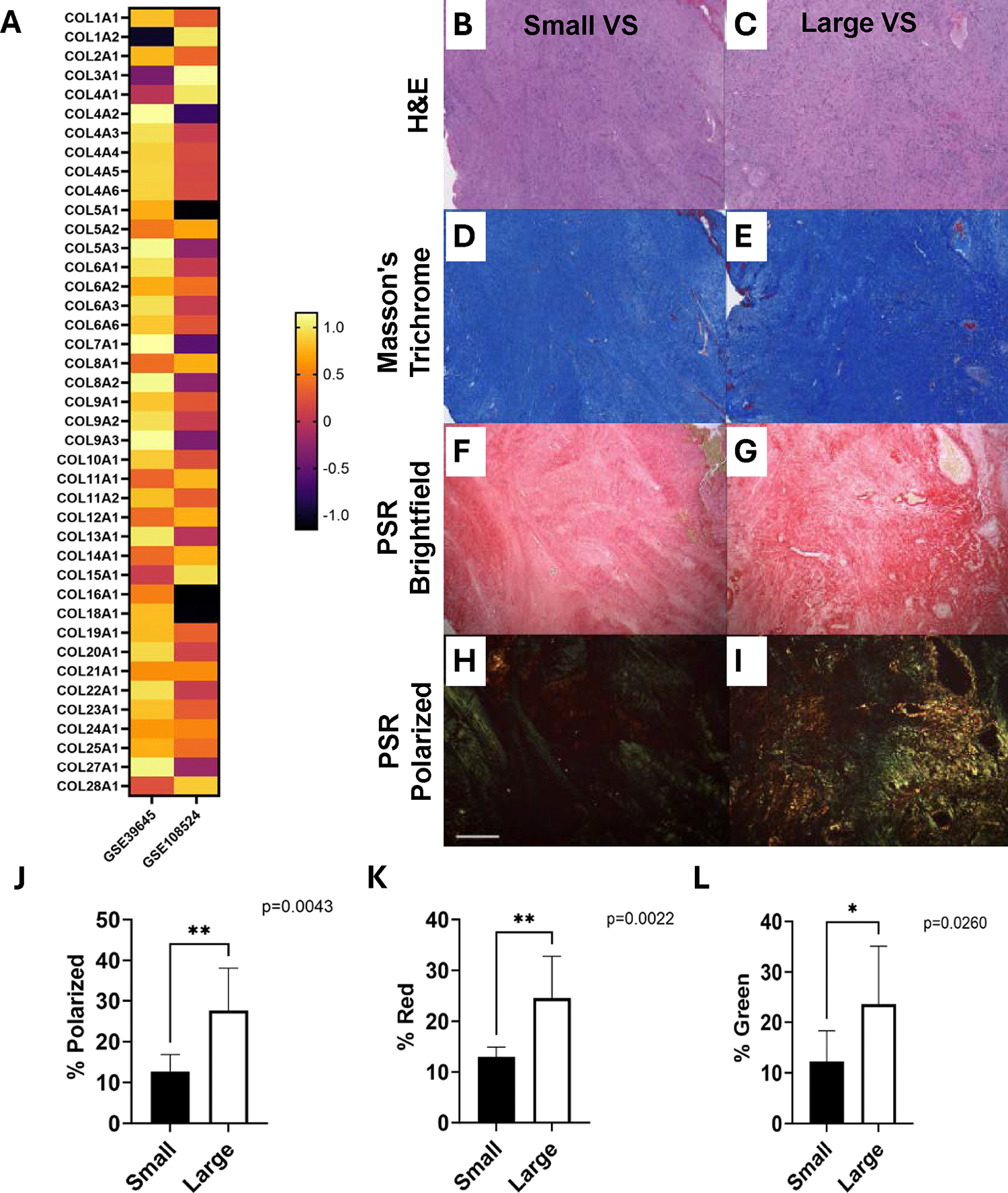
Collagen is increased in vestibular schwannoma (VS). (A) Expression of collagen from microarrays. (B–I) Representative staining of small (left) vs large VS (right). (J–L) Quantification of total, mature (% red), and immature collagen (% green). PSR, picrosirius red.

Collagen is an integral component of the TME and ECM, and dysregulated deposition of collagen fibers is implicated in the progression of desmoplastic tumors.^23^ To better understand the type of collagen in VS, we examined mRNA expression of collagen between VS (n = 38) and normal tissue (n = 12) using publicly available datasets (GSE39645 and GSE108524). We discovered that most collagen subtypes are upregulated in VS compared to normal nerve (Figure 1A). This overexpression across multiple collagen subtypes suggests a potential role of collagen in VS pathology and was the basis for further investigation of collagen in the TME of VS.

### Increased collagen abundance in larger tumors

To examine the deposition and topology of collagen, we retrospectively analyzed 11 patients with sporadic VS. Average maximal tumor diameter was 2.80 cm (range, 1.50–4.00 cm, 82% male, average age 44 ± 11 years) (Table 1). With the goal of studying the collagen composition in relation to VS progression, the cohort was split into two groups by tumor size, (3.5 vs 2.0 cm, *P* < .001). H&E staining provided general insights to tissue morphology (Figure 1B and C), while Masson’s Trichrome offered enhanced visualization of collagen (Figure 1D and E). PSR stain, when combined with brightfield and polarized light microscopy, allowed for a detailed assessment of collagen density and subtype differentiation (Figure 1F–I). This approach revealed that, compared to small VS, larger tumors had a significantly higher polarization content (27.6% vs. 12.7%, Mann-Whitney *U*-test *P* = .0043), indicative of an overall greater collagen density (Figure 1J). Additionally, PSR polarized images were analyzed to determine the density of thick fibers (% red) depicting mature collagen Type I and thin fibers (% green) representing immature collagen Type I or mature collagen Type III. This distinction between thin and thick fibers revealed that larger tumor exhibited significantly higher density of both thick fibers (24.5% vs 13.0%, *P* = 0.0022) and thin fibers (23.6,% vs 12.3%, *P* = 0.026) when compared to small VS (Figure 1K and L). This approach emphasizes the increased collagen accumulation and maturation in larger tumors.

### Collagen is More Disorganized during VS Progression

**Figure 2.**
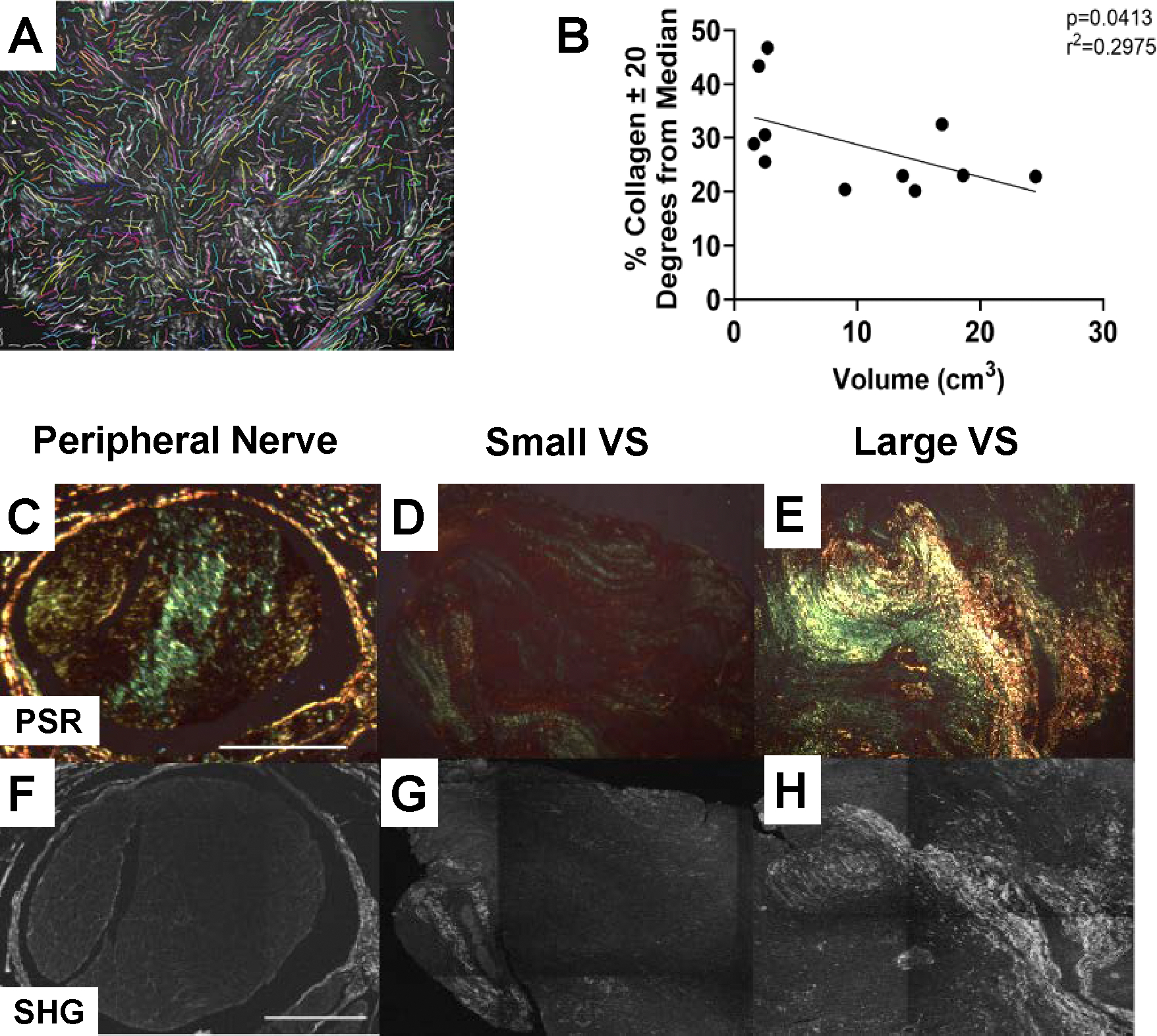
Disorganization of collagen during vestibular schwannomas (VS) progression. (A) CT-FIRE output. (B) Correlation between tumor volume and collagen alignment. (C–H) picrosirius red (PSR) polarized (top) and second harmonic generation (SHG) (bottom) of peripheral nerve, small VS, and large VS.

Not only is collagen composition important, but also collagen architecture as it can enhance the invasive potential of tumor. Using CT-FIRE, an automated quantitative collagen fiber analysis software, we quantified several collagen fiber characteristics, including fiber number, width, length, straightness, and angle using polarized light images of PSR-stained VS sections (Figure 2A). Output is color-coded representing various collagen fibers. Quantification was performed on n = 11 tumors, with 3 to 5 ROIs analyzed from each tumor. Importantly, the percent of collagen that is aligned ±20° from the median, a surrogate marker for collagen architecture disorganization, is negatively correlated with tumor volume by Person’s correlation test (*r*^2^ = 0.2975, *P* = .0413, Figure 2B). This suggests that larger tumors exhibit more misaligned collagen organization.

There was no significant correlation between tumor volume and fiber number, width, length, or fiber straightness (Data not shown). Second harmonic generation (SHG) microscopy confirmed similar fiber architecture as seen in PSR by detecting nonlinear signals generated by collagen, in normal peripheral nerve (Figure 2C and F), small VS (Figure 2D and G) and large VS (Figure 2E and H). Taken together, our results demonstrate that as VS progresses, collagen is remodeled into a more disorganized manner, and we can visualize collagen architecture in VS tissue using specialized imaging techniques.

### Collagen Type I is Enriched in VS

**Figure 3.**
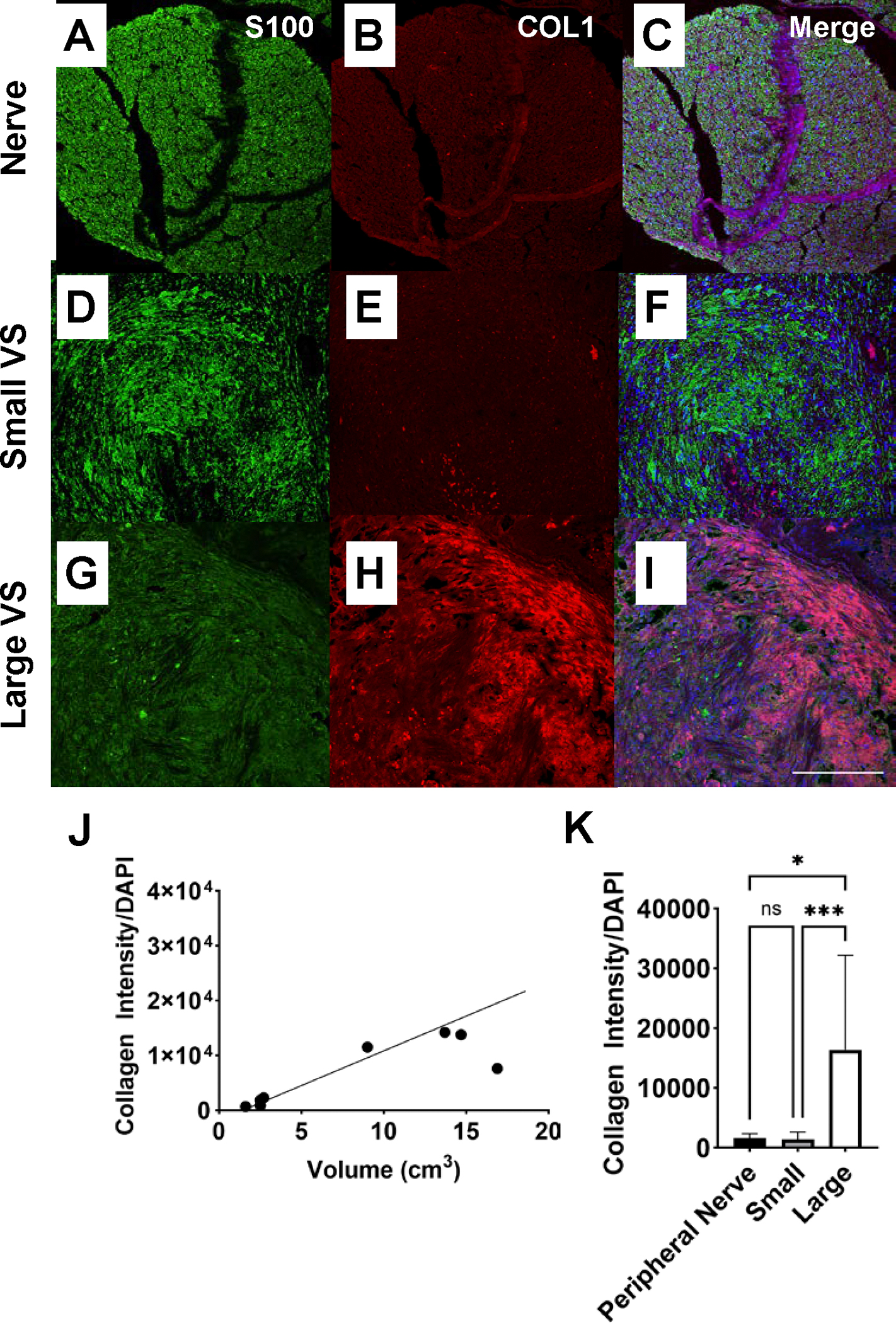
Collagen I in vestibular schwannomas (VS). (A–I) Collagen-1 immunofluorescence (DAPI, blue; S100b, green; Collagen I, red). (J) Collagen I correlates with tumor volume. (K) Collagen-1 is more abundant in large VS.

Collagen Type I, the most abundant fibrillar collagen subtype in the TME of many cancer types, has been shown to be upregulated in VS and may play a role in mediating tumor cell-ECM interactions.^7^ To further investigate the type of collagen deposited in VS, immunofluorescence against collagen Type I was performed in peripheral nerve (Figure 3A–C), small VS (n = 4, representative images shown in Figure 3D–F) and large VS (n = 5, representative images shown in Figure 3G–I). Sections were co-stained with DAPI to label cell nuclei, S100b to label neural-crest origin cells (Figure 3A, D, and G), and Collagen-1 (Figure 3B, E, and H). This demonstrated a substantial proportion of the TME was positive for collagen type I (Figure 3C, F, and I). We also demonstrate a significant and positive correlation between tumor volume and collagen intensity relative to the number of schwannoma cells (S100+) in each histological section (*r*^2^ = 0.6524, Pearson’s correlation test, *P* = .0085) (Figure 3J). Additionally, Collagen **I** intensity normalized to tumor surface area, as measured by the intensity of S100b positivity, was significantly increased by over 11-fold in large VS compared to small tumors (mean fluorescence intensity normalized to cell count 16395 vs 1430, 1-way ANOVA with Tukey’s post hoc test, *P* = .0002) and to peripheral nerve control (1714, *P* = .0275) (Figure 3K). These results demonstrate that Collagen I is a major collagen subtype that is upregulated and deposited in the TME during VS progression.

## Discussion

Utilizing specialized microscopy and an automated quantitative assessment of collagen architecture, we demonstrate that collagen deposition and architecture can be accurately quantified in VS tissue. Furthermore, we demonstrated that collagen is increasingly deposited as VS progresses, with large tumors demonstrating increased collagen fibers that are more disorganized compared to small tumors. Additionally, collagen type 1 is a dominant component of the VS TME and may represent a target for new pharmacotherapies.

During desmoplastic tumor progression, the TME undergoes constantly remodeling in response to physical and biochemical cues, with continuous degradation and deposition of ECM being a key part of this process. As corroborated in our study, collagen proteins, a major component of the ECM, have been shown to influence the behavior of the cells around it. This effect on behavior includes alterations in metabolism,^24^ metastatic and invasive potential,^25^ resistance to targeted therapies^26–28^ and is related to overall poor outcomes.^29^

In the context of VS, Shi et al reported that upregulation of collagen is associated with poor outcomes following stereotactic radiosurgery utilizing a similar microarray database as our study (GSE141801, n = 67 patients).^30^ In a preclinical cerebellopontine schwannoma mouse model, treatment with an angiotensin-receptor blocker (losartan) as an anti-fibrotic through inhibition of IL-6/STAT3 and TGF-β, reduced the amount of collagen I and prevented tumor-induced hearing loss.^22^ Retrospective analysis of 45 VS patients with hypertension showed a lower risk of hearing loss in those taking losartan. However, in a later study of 79 patients with VS and hypertension, of which 29 were treated with losartan, there was no significant advantage in terms of slowing tumor growth or preventing VS-associated hearing loss.^21^ While these two studies are commendable for repurposing widely available drugs, the conflicting findings underscore the need for more research focused on targeting specific pathways involved in collagen deposition. Given the complexity of collagen deposition, losartan may not be the optimal choice for VS, which is why our study aims to highlight the deposition and organization of collagen to inform future research efforts.

Most published studies utilize H&E with the addition of immunofluorescence or routine histological stains (such as Masson’s Trichrome) to assess the ECM structure.^3,22,31^ While these methods provide basic information about the ECM, they provide limited information on the maturity or structure of collagen proteins. By utilizing PSR with polarized microscopy in VS, our study demonstrates, for the first time, an increase in both mature and immature collagen fibers during VS progression, suggesting significant and continued ECM remodeling. This deposition may be due to activation of profibrotic signaling pathways that lead to collagen production in both schwannoma cells and activated fibroblasts, representing potential therapeutic targets for therapies such as anti-TGF-b antibodies (Fresolimumab) or collagen-targeting nanoparticles for phothermal therapy. The isolation of collagen type 1 in our study is crucial for designing specific pharmacotherapies for the treatment of VS such as collagenase treatment^32^ or inhibiting collagen cross-linking.

Moreover, the architecture of the collagen, and not simply the abundance, has also been implicated in the pathogenesis of aggressive tumor subtypes.^33–36^ In breast cancer, collagen organization was linked to cellular invasion of nearby tissue, with studies on glioblastoma reporting that disorganized collagen architecture was associated with worse patient survival.^36^ This is corroborated in our study by demonstrating that large VS, which have significantly worse outcomes than small VS,^37,38^ have disorganized collagen architecture. As VS progresses, greater inflammation, and increased amounts of collagen I-producing cells, including schwannoma cells and activated fibroblasts, may lead to increased deposition of ECM.^9,39^ However, the degradation of collagen by matrix metalloproteases plays an equally significant role in the development of a fibrotic TME. Moreover, upregulation of MMP-9 enhanced schwannoma cell migration and invasion in vitro.^20^ In VS that underwent subtotal resection, there was an elevated expression and secretion of MMP-14 by schwannoma cells.^40^ Other published studies have demonstrated that MMP-2, MMP-9, and MMP-14 are upregulated in aggressive, large, and adherent VS, which could be responsible for the apparent degradation and subsequent deposition of collagen.^20,41^

The highly heterogenous nature of the collagen in the VS TME may lead to altered mechanotransduction of signals from the ECM to tumor cells or from schwannoma cells to extrinsic environment, in addition to serving as a physical barrier to immune cell infiltration including macrophages and T cells.^42^ Moreover, macrophages, which make up the majority of the non-schwannoma cells in VS, develop into an M2-polarized phenotype due to the cytokines secreted in an irregular and dense ECM.^17,43^ These M2 macrophages (CD163+) have been shown to correlate with VS growth, further linking VS progression with disorganized collagen deposition and macrophage polarization.^44^ By using CT-FIRE with PSR, we performed an in-depth analysis of collagen organization in VS slides, opening the door for future studies examining various clinical parameters related to collagen architecture.

Outside of cellular behavior, the deposition of collagen impacts the overall biomechanical properties of the tumor. These properties, such as biomechanical stiffness that can be noninvasively measured through magnetic resonance elastography,^3^ have direct effects on the outcomes of patients undergoing surgical resection, such as firm meningiomas exhibiting worse postoperative outcomes.^45^ Our recent work established that stiff VS, characterized by increased collagen, also demonstrated poor postoperative outcomes including greater incidence of subtotal resections, leading to worse facial nerve function.^3^ Targeting collagen may have the potential to improve patient outcomes by addressing both the molecular pathways and overall biomechanical properties of the VS TME.

This study has several limitations. One main limitation is the small sample size and retrospective nature, which affects the broad generalizability of our findings. Future studies should include a larger prospective cohort to measure the association between collagen architecture and clinical outcomes in VS. Moreover, our clinical analysis is limited to tumor size and association with collagen microarchitecture. However, we understand that the temporal aspect of progression is not well characterized by size of the tumor at the time of surgery, as tumors may reach a large volume at different times due to the unpredictable growth rate. Additional clinical features to analyze would be the presence of adhesions to the facial nerve, the presence of cystic components, and the evaluation of tumor consistency. Finally, our analysis is limited to collagen type 1. Given the abundance of other types of collagens in VS on the transcriptomic and proteomic level, future investigations should expand to other collagen subtypes in VS.

In conclusion, our study utilizes various and novel quantitative modalities to explore the correlation between collagen architecture, abundance, and collagen subtype with VS progression. We demonstrate that as VS grows, the TME is characterized by dense and irregular collagen deposition, with collagen type 1 predominating. Our study highlights collagen deposition as a potential key modulator in aggressive VS, and future studies should be performed to analyze the effect of collagen-targeting pharmacotherapies.

## Figures and Tables

**Table 1.** Demographics of Patients in the Study

	Total (n=11)	Small (n=5)	Large (n=6)
Age (years ± SD)	44 ± 11	51 ± 10	38 ± 9
Male (n, %)	9 (82%)	4 (67%)	5 (83%)
Maximum diameter (cm ± SD)	2.8 ± 0.9	2.3 ± 0.5	3.5 ± 0.4
Volume (cm^3^ ± SD)	9.9 ± 8.2	2.3 ± 0.5	16.2 ± 5.2
Cystic n, (%)	5 (45%)	3 (60%)	2 (33%)
Gross/near-total n, (%)	8 (73%)	5 (100%)	3 (50%)
